# The Same yet Different: Oral and Silent Reading in Children and Adolescents with Dyslexia

**DOI:** 10.1007/s10936-022-09856-w

**Published:** 2022-03-04

**Authors:** Madelon van den Boer, Loes Bazen, Elise de Bree

**Affiliations:** Research Institute of Child Development and Education, University of Amsterdam, PO Box 15780, 1001 NG Amsterdam, The Netherlands

**Keywords:** Dyslexia, Oral reading, Silent reading, Reading fluency

## Abstract

Dyslexia is characterized by poor word reading. In research, education, and diagnosis, *oral* reading is commonly assessed, and outcomes are generalized to *silent* reading, although similarities and differences between oral and silent reading are poorly understood. We therefore compared oral word reading, oral text reading and silent text reading. Children (*n* = 40; aged 8–11) and adolescents (*n* = 54; aged 14–18) with dyslexia, and typical readers (*n* = 18, and *n* = 24 respectively), read a word-list and an age-appropriate text aloud, and silently read a text including instructions for simple tasks. Whereas oral and silent reading fluency were comparable for children, silent reading was more fluent than oral reading for adolescents. Importantly, the silent reading deficit of children and adolescents with dyslexia was as large as in oral reading or larger, highlighting the need for a focus on both reading modes in research, diagnosis and treatment of dyslexia.

## Introduction

Developmental dyslexia is characterized by difficulties with accurate and/or rapid reading and/or spelling of words (American Psychiatric Association, 2013; Lyon et al., 2003; Peterson & Pennington, 2015). Reading accuracy, referring to correct phonological decoding, is relatively easy to acquire in orthographies that have a relatively transparent association between phonemes and graphemes (i.e., (semi-)transparent orthographies; Seymour et al., 2003). Accuracy outcomes approach ceiling after only a few months of reading instruction (e.g., Wimmer & Hummer, 1990) and even poor readers are able to decode words at the end of first grade (e.g., de Jong & van der Leij, 2003; Klicpera & Schabmann, 1993; Landerl & Wimmer, 2008). In contrast, word reading fluency deficits are persistent (e.g., de Jong & van der Leij, 2002; Moll et al., 2019). Reading fluency is thus the reading measure that differentiates most adequately between good and poor readers. Reading fluency can be defined in a number of ways (e.g., Kuhn et al., 2010). In line with the definition of dyslexia, we adopt a relatively narrow definition of reading fluency with a focus on word recognition, that is the number of syllables that can be correctly identified within a given amount of time.

To measure potential reading difficulties in the context of diagnosing dyslexia, it is common practice to administer tests that require reading words *aloud*. Although this measurement of reading fluency corresponds to the definition of dyslexia, it is at odds with daily experiences that more often require silent reading fluency, and reading of texts rather than words. This issue is especially relevant since dyslexia is a lifelong condition, and even though dyslexia is often diagnosed in elementary school, there are students who receive a diagnosis of dyslexia only later on in their school career, around Grade 7/8 or even later (e.g., Bazen et al., 2020; Torppa et al., 2015). At this (st)age, silent reading, text reading fluency, and comprehension of text are central to the school curriculum. At that (st)age the role of linguistic comprehension in reading comprehension has been found to be larger than the role of decoding skills; accordingly, individuals with dyslexia are not necessarily impaired in reading comprehension (e.g., Bazen et al., 2020; García & Cain, 2014; Lonigan et al., 2018). Nevertheless, text reading fluency has been found to predict reading comprehension after controlling for decoding and linguistic comprehension (Kershaw & Schatschneider, 2012). Measuring text reading fluency, in both the oral and silent reading mode, is therefore essential in terms of assessment and support for dyslexia after the initial stage of learning to read.

However, silent reading is usually not considered when diagnosing or treating individuals with dyslexia. In contrast, accommodations offered to compensate the effect of the reading and spelling disorder do tend to focus on silent reading, such as additional time to complete exams and text-reading aloud software (Buzick & Stone, 2014; Lovett & Leja, 2013; Wood et al., 2018). The tendency to examine oral reading at the expense of silent reading is not specific to dyslexia, but extends to reading research and practice in general (Share, 2008). As a result, we know very little about similarities and differences between oral and silent reading, in terms of performance and precursors, but also (diagnostic) measurements and effects of training or accommodations. In other words, we do not know whether the accommodations offered for dyslexia are necessary or sufficient. Moreover, we do not know if oral and silent reading can diverge in individuals with dyslexia. If reading deficits can differ in oral and silent reading, it would be important to include both oral and silent reading in diagnosis and treatment for dyslexia. In the current study we therefore examine whether oral and silent reading fluency are the same or different for both children and adolescents with and without dyslexia.

The reason that reading fluency is most often examined in the oral reading mode is straightforward: Oral reading of words and texts allows the examiner to monitor reading processes and ensure that the material is read as intended, whereas accuracy and speed during silent reading cannot be directly observed. Measures of silent reading fluency often include a decision component, such as semantic categorization, sentence verification or lexical decision (e.g., Bar-Kochva, 2013; Kim et al, 2011; Zeguers et al., 2011). Asking children to, respectively, determine whether a certain word (e.g., ‘dog’) belongs to a certain category (e.g., ‘animal’), indicate whether a given sentence (e.g., ‘The grass is blue’) is correct or incorrect, or decide whether a letter string is or is not an existing word, obviously appeals to language and comprehension skills in addition to decoding skills, and thus does not purely reflect reading fluency. Previous studies that have compared oral and silent reading fluency measured in similar ways included typical readers and have indicated that there are in fact differences between the two reading modes. Oral and silent reading seem to reflect distinct, but highly related skills (Kim et al., 2011), and silent reading tends to proceed faster than oral reading (e.g., Krieber et al., 2017; McCallum et al., 2004). In adults, silent reading has been found to be almost twice as fast as oral reading (Ciuffo et al., 2017). Moreover, silent reading speed increased throughout early adulthood, whereas oral reading speed reached ceiling by adolescence.

The reasons behind these differences between oral and silent reading fluency are still poorly understood. To our knowledge, there is, for example, no (developmental) theoretical model of reading fluency that includes both oral and silent reading. Two types of studies do provide some insight into potentially relevant differences. Van den Boer et al. (2014) studied the differences across reading modes by assessing the relations of oral and silent reading fluency with underlying cognitive skills in a sample of fourth graders. Oral reading was found to relate more strongly to rapid naming, the ability to quickly name highly familiar stimuli such as digits. In contrast, phoneme awareness, the ability to identify and manipulate phonemes in spoken language, was related to both silent and oral reading. The findings indicate, in line with models of speech production (e.g., Levelt, 1992), that selecting and accessing spoken word forms is common to both oral and silent reading. In oral reading, however, an additional step is required, that is the production and articulation of verbal output. This additional production and articulation of verbal output takes time and as such could hinder oral reading fluency.

Other studies have used eye tracking to look closely at online reading processes during oral and silent reading (Ashby et al., 2012; Vorstius et al., 2014). Vorstius et al. (2014) found that differences between oral and silent reading in children are due to additional processing demands of speech planning, articulation and eye-voice coordination during oral reading. In addition, Ashby et al. (2012) found that in adults, parafoveal information (i.e., information just outside the current fixation of the eyes), affected silent reading more than oral reading. This finding indicates that the resources allocated to the additional processing steps during oral reading deplete resources available for parafoveal processing, thus slowing down reading.

There is only one study that examined oral and silent reading fluency for individuals with dyslexia. Gagliano et al. (2015) studied silent reading in adults with an official diagnosis of dyslexia and skilled adult readers, to gain insight into appropriate means of assessing reading difficulties. Since silent reading is the primary mode of reading for adults, Gagliano et al. (2015) argue that understanding and measuring silent reading performance is of paramount importance in diagnosing dyslexia at this age. The authors directly tested whether differences between oral and silent reading are the same or different for Italian adults with and without dyslexia. They used a new, ecologically valid task to assess silent reading fluency. Silent reading fluency was defined as the speed with which adults were able to understand and follow written instructions. Specifically, the adults had to read a text that included instructions to perform simple tasks (e.g., ‘knock twice on the table’). The reader’s execution of these simple tasks was observed to ensure careful reading. In addition, the total time the reader needed to complete the text was calculated to determine the reader’s silent reading fluency. Performance on the silent reading task was compared to oral reading fluency on an age-appropriate short text. As expected, the results showed that adults without dyslexia read faster than adults with dyslexia on both the oral and silent reading task. Importantly, however, the mean reading speed of adults with dyslexia was found to be 25 percent higher in silent as compared to oral reading, whereas fluent readers were found to read up to 62 percent faster silently than orally. Their results thus indicated that the difference between oral and silent reading fluency is much smaller for adults with dyslexia compared to adults with average to good reading skills. In other words, the difference between oral and silent reading appeared to increase with reading skill.

In the current study, we aimed to add to the existing data on the difference between oral and silent reading fluency for individuals with dyslexia. We focused on both children and adolescents with dyslexia, to extend the findings of Gagliano et al. (2015), who focused only on adults. It is important to focus on children and adolescents, because diagnosis and treatment of dyslexia most often occur earlier than adulthood. We need to know whether the reading deficit for individuals with dyslexia is the same, larger, or smaller in silent compared to oral reading, in order to make informed decisions about the role of oral and silent reading in both diagnosis and treatment. If oral and silent reading fluency differ, and especially, if the deficit of individuals with dyslexia differs in oral as compared to silent reading, both oral and silent reading should be targeted in diagnostic examinations and interventions for dyslexia.

Because there is so little theoretical and empirical evidence on the differences between oral and silent reading and the development thereof, few specific hypotheses could be deduced from the literature. Similar to Gagliano et al. we might expect the difference between oral and silent reading to be smaller in adolescents with dyslexia compared to typically developing peers, indicating a relatively strong impairment in silent as compared to oral reading. In contrast, based on findings of van den Boer et al. (2014) on the stronger involvement of rapid naming in oral compared to silent reading, we might expect stronger deficits in oral as compared to silent reading, as rapid naming tends to be impaired in adolescents with dyslexia (e.g., Bazen et al., 2020; Torppa et al., 2015).

For the children, who are less experienced readers, the expectation is even less clear. The difference between reading aloud and silently could be less pronounced, for both children with and without dyslexia. Reading development starts out as a social process (Prior & Welling, 2001), since children start by being read to and by reading aloud. Only after a few years of reading instruction does the focus start to shift to silent reading as the most common, best developed form of reading. As a result, in the early years of reading development, children who mainly read aloud, might read similarly when reading silently, resulting in no difference between the reading modes.

## Study 1—Method

### Participants

Participants in this study were 40 third through fifth grade children with dyslexia. They were all students in mainstream education at regular primary schools, and were all fluent speakers of Dutch, although two children spoke another language at home. Participants needed to have an official diagnosis of dyslexia. No further inclusion or exclusion criteria were applied. In the Netherlands, dyslexia is diagnosed by specialized health care professionals following specific protocols (NRD, 2013; SDN, 2016). The main criterion for dyslexia is a score in the lowest 10% on a standardized measure of word reading and/or spelling (SDN, 2016). Furthermore, for children in primary school a specific protocol for diagnosing dyslexia is most often adhered to (NRD, 2013), which additionally requires that if spelling is in the lowest 10%, reading performance is at least in the lowest 16%, and phonological processing is impaired as indicated by performance in the lowest 10% on letter-sound associations, phonological awareness, and/or rapid automatized naming. In all cases these deficits need to occur despite adequate educational opportunities, and in the absence of sensory deficits or general intellectual disabilities. Furthermore, comorbid disorders, such as attention deficit (hyperactivity) disorder or developmental language disorder, are a contraindication for a diagnosis of dyslexia, but only if these comorbid disorders are severe and untreated. If comorbid disorders have been treated and dyslexia can thus be regarded the primary disorder, dyslexia can be diagnosed in the presence of other disorders.

We also included a group of typical readers consisting of 18 third and fourth graders, who were all average to good readers and native speakers of Dutch. Information about the participants is presented in Table 1. The two groups did not differ in age and gender. As expected, the groups did differ in reading ability, with typical readers reading more words correctly per minute than children with dyslexia.

**Table 1:** Descriptives study 1

	Children with dyslexia (*N* = 40)	Typical readers (*N* = 18)	*t*/*χ*^2^	*p*
*n* boys/girls	24/16	13/5	0.803	.370
Age	9;9	9;8	0.137	.892
Word reading fluency	35.15 (10.41)	58.76 (12.18)	7.446	< .001
Oral reading fluency^a^	1.72 (.60)	3.01 (.72)	7.144	< .001
Silent reading fluency^a^	1.73 (.71)	3.14 (.77)	6.831	< .001

^a^In syllables per second

### Procedure

Children with a formal diagnosis of dyslexia were recruited from health care centers where they were receiving treatment for their reading and spelling difficulties. Typical readers were partly recruited via the children with dyslexia, by asking them to bring a friend without reading difficulties to the center for testing. In addition, typical readers were recruited from Grades 3 and 4 of one primary school in the Netherlands. A letter explaining the study was distributed among parents of both groups of children. When the letter was returned with parents’ informed consent, children were included in the study. They were tested individually at the health care center or at their home. The session, including the two text reading tasks, the standardized word reading task, and several tasks for another study, lasted about 30 min. The oral and silent text reading tasks were presented in a counterbalanced order. This study was approved by the ethics committee of the university (number: 2016-CDE-6725).

### Materials

**Oral word reading** To assess oral word reading fluency, we used the standardized One Minute Test (*Eén Minuut Test;* Brus & Voeten, 1995; *r* = 0.89–0.97). Children were asked to read aloud as quickly and accurately as possible a list of 116 words of increasing difficulty. The score consisted of the number of words read correctly within one minute.

**Oral text reading** To assess oral text reading fluency, a text was selected from an old version of curriculum-based measures for text reading (AVI; Visser et al., 1996). We used an old version to make sure we did not interfere with the curriculum-based measurements at school. The selected text was designed to assess reading by the end of Grade 3. We chose this text to ensure that the text was age-appropriate, but not too difficult for the poorer readers in the sample. The text consisted of 202 words, 278 syllables. Children were asked to read the text aloud as quickly and accurately as possible. The score consisted of the time in seconds it took children to read the entire text, converted to the number of syllables read per second. Different from standard administration of this task, errors in reading were not scored and not taken into account in the score to minimize differences with silent text reading, for which errors in reading cannot be observed.

**Silent text reading** Following the procedure of Gagliano et al. (2015), a text was designed for this study to assess silent reading fluency. We created an age-appropriate text, that was similar in difficulty to the oral reading task. Children were asked to read the text (246 words, 352 syllables) silently as quickly and accurately as possible. To ensure and check careful reading, the text included instructions for six simple tasks (i.e., clap your hands, pick up the yellow block). To familiarize children with this procedure a practice text (55 words, 79 syllables) was presented, including 2 simple tasks. The experimenter registered whether the six tasks were performed. Performance of the final task indicated that the child had finished reading the text. The score for silent text reading consisted of the time in seconds it took children to read the entire text, converted to the number of syllables read per second. To illustrate the task, a translation of the first paragraph of the text is presented here:

The reading task with assignments is going to start. The first assignment is that you pick up the yellow block. Well done. Pay attention, you don’t always have to use the blocks. At the end of the sentence, for instance, you pronounce the word ‘banana’ out loud. Excellent. We will keep track of whether you have done so, so you can repeat the word once more. Go ahead and say it. Well done!

### Data Analysis

To examine whether oral and silent reading fluency were the same or different for children with and without dyslexia, we used a 2 (mode: oral, silent) × 2 (group: with and without dyslexia) repeated measures ANOVA, as well as the correlation between oral and silent reading fluency. Because the measure of oral reading only reflected reading fluency, whereas the measure of silent reading also included performance of simple tasks, the RM ANOVA results should be interpreted with caution. Nevertheless, this method of analysis best fitted the research questions, which mainly concerned the difference between oral and silent reading (i.e., main effect of reading mode) and a comparison of this difference across groups (i.e., interaction of reading mode x group). In addition, we examined the correlation of oral and silent reading fluency with a standardized measure of oral word reading fluency (Brus & Voeten, 1995), to see if this commonly used standardized measure adequately reflected oral and/or silent text reading fluency.

## Study 1—Results

First, we checked whether the silent reading task was performed as intended. One child with dyslexia performed only two of the six simple tasks included in the text. The score of this child was therefore unreliable and was left out of the analyses. All other participants performed four or more of the six tasks included in the silent reading text correctly. We report the analyses that included all those participants, but the results were the same when we only included children who performed all six tasks correctly. There were no outliers (i.e., scores more than three standard deviations from the mean) and the scores were normally distributed.

**Fig. 1:**
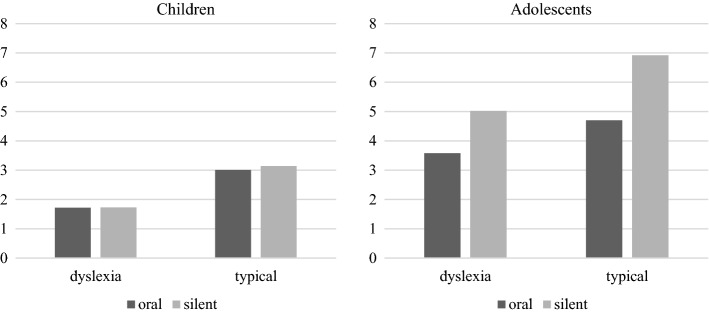
Word reading results (in syllables per second) per group and reading mode

The descriptive statistics are presented in Table 1 and Fig. 1. As expected, children with dyslexia read texts significantly more slowly than typical readers (*F*(1, 55) = 52.16, *p* < 0.001, η_p_^2^ = 0.487). However, fluency of oral and silent reading did not differ from each other, neither for the children with dyslexia, nor for the typical readers, as shown by the absence of the main effect of reading mode (*F*(1, 55) = 1.98, *p* = 0.165, η_p_^2^ = 0.035), and the absence of an interaction between reading mode and group (*F*(1, 55) = 1.19, *p* = 0.280, η_p_^2^ = 0.021).

Oral and silent text reading fluency were very strongly correlated for the total group of participants (*r* = 0.922, *p* < 0.001), as well as separately for children with dyslexia (*r* = 0.862, *p* < 0.001) and typical readers (*r* = 0.840, *p* < 0.001). In addition, both oral and silent reading correlated strongly with performance on the standardized oral word reading test for the total group of participants (*r* = 0.912, *p* < 0.001, and *r* = 0.879, *p* < 0.001 respectively), as well as separately for children with dyslexia (*r* = 0.878, *p* < 0.001, and *r* = 0.814, *p* < 0.001 respectively), and typical readers (*r* = 0.745, *p* < 0.001, and *r* = 0.701, *p* < 0.001 respectively). These findings indicate that oral and silent reading fluency do not differ for children in primary school, and that children with dyslexia show equally strong deficits in both reading modes.

## Study 2—Method

### Participants

Fifty-four students with dyslexia were included in this study. Their performance was compared to that of 24 typical readers. They were all in Grade 10 of mainstream education at a regular secondary school. Students with dyslexia all had an official diagnosis of dyslexia, based on the criteria described for the participants of Study 1. Because some participants were diagnosed several years ago, an additional criterion was that they obtained a score within the lowest 10% on a standardized word and/or pseudoword reading fluency test at the time of testing (One Minute Test: Brus & Voeten, 1995; and Klepel: van den Bos et al., 1994). Typical readers were only selected if they scored above the tenth percentile on both of these reading tests. All participants were native speakers of Dutch. Information about the participants is presented in Table 2. The two groups did not differ in age and gender. They did, of course, differ in reading ability, with typical readers reading more words correctly per minute than students with dyslexia.

**Table 2:** Descriptives study 2

	Adolescents with dyslexia (*N* = 54)	Typical readers (*N* = 24)	*t*/*χ*^2^	*p*
*n* boys/girls	29/25	11/13	0.412	.521
Age	16;6	16;3	0.620	.537
Word reading fluency	71.48 (11.04)	96.67 (11.58)	7.446	< .001
Oral reading fluency^a^	3.58 (.56)	4.70 (.54)	8.134	< .001
Silent reading fluency^a^	5.02 (1.20)	6.92 (1.20)	6.332	< .001

^a^In syllables per second

### Procedure

Adolescents with a formal diagnosis of dyslexia were recruited from nine high schools throughout the Netherlands. Adolescents with dyslexia introduced typical readers to the study, by nominating a friend without reading or spelling difficulties for testing. Additional students in both groups were recruited through the second authors’ network and social media. Active informed consent was obtained from both parents and adolescents prior to testing. The tests included in the current study were part of a larger test battery (see Bazen et al., 2020), and were both administered during the first of three sessions that lasted 45–60 min each. Testing took place in a quiet room within the school, at the university or at the participant’s home. The study was approved by the ethics committee of the university (number: 2015-CDE-4749).

### Materials

**Oral word reading** To assess oral word reading fluency we used the same task as for Study 1.

**Oral text reading** To assess oral text reading fluency, an excerpt from the text ‘Domoren verpesten gehoor’ [*Fools damage hearing*] by Rivka Bijl was selected from a final exam Dutch reading skills, prevocational education 2010–1 (CITO, 2010). The text consisted of 243 words (397 syllables), and was age-appropriate in both level and topic. Similar to Study 1, participants were asked to read the text aloud as quickly and accurately as possible. The score consisted of the number of syllables read per second.

**Silent text reading** Similar to Study 1 a text was designed for this study to assess silent text reading fluency, following the procedure of Gagliano et al. (2015). The text was similar to the text of Study 1 in content as well as the tasks included, but different in level (i.e., included more difficult words). The text for the adolescents consisted of 404 words, 606 syllables, and was preceded by a practice text of 57 words and 80 syllables. The score consisted of the number of syllables read per second.

### Data analysis

Data analysis was the same as for Study 1.

## Study 2—Results

Silent text reading speed of one typical reader was found to be more than three standard deviations above the group mean and was excluded from the analyses. Oral text reading speed was missing for one typical reader. All participants performed four or more of the six simple tasks included in the text correctly during silent reading. We report the analyses that included all those participants, but the results were the same when we excluded the three participants who skipped one or two tasks from the analyses. The scores on both reading tasks were normally distributed.

The descriptive statistics are presented in Table 2 and Fig. 1. As expected, adolescents with dyslexia read texts significantly more slowly than typical readers during both oral and silent reading (*F*(1,74) = 52.73, *p* < 0.001, η_p_^2^ = 0.416). In addition, silent text reading was faster than oral text reading (*F*(1,74) = 239.93, *p* < 0.001, η_p_^2^ = 0.764). The significant interaction effect indicated that this difference was smaller for adolescents with dyslexia than for typical readers (*F*(1,74) = 12.03, *p* = 0.001, η_p_^2^ = 0.140). Please note, however, that the interaction effect is partly affected by the overall difference in reading times across groups. When compared to their own oral reading rate, silent reading was 47.2% faster for typical readers, and 40.2% faster for adolescents with dyslexia. Follow up t-test indicated that these differences were significant for both typical readers (*t*(21) = 10.998, *p* < 0.001), and adolescents with dyslexia (*t*(53) = 11.274, *p* < 0.001).

Oral and silent text reading performance were strongly correlated for the total group of participants (*r* = 0.784, *p* < 0.001), and moderately when analyzed separately for adolescents with dyslexia (*r* = 0.646, *p* < 0.001) and typical readers (*r* = 0.676, *p* < 0.001). Correlations with performance on the standardized oral word reading test differed for oral and silent reading, as well as per group. For the total group of participants, the correlation between oral word reading and oral text reading (*r* = 0.823, *p* < 0.001) appeared to be somewhat higher than with silent text reading (*r* = 0.714, *p* < 0.001). However, this pattern differed for the groups separately: for the adolescents with dyslexia, correlations between oral word reading and oral and silent text reading were found to be similar and moderate (oral: *r* = 0.650, *p* < 0.001; silent: *r* = 0.580, *p* < 0.001; Fisher’s *z* = 0.799, *p* = 0.212). In contrast, for typical readers the correlation with oral reading was stronger than with silent reading (oral: *r* = 0.662, *p* = 0.001; silent: *r* = 0.283, *p* = 0.191; Fisher’s *z* = 2.597, *p* < 0.001). These findings indicate that silent reading is more fluent than oral reading in adolescents with and without dyslexia, but for typical readers the difference between oral and silent reading was larger than for adolescents with dyslexia. In other words, adolescents with dyslexia show deficits in both oral and silent reading, but on average, their deficit in silent reading fluency is somewhat larger than in oral reading fluency.

## General Discussion

In the current study we examined differences between oral and silent text reading fluency for children and adolescents with and without dyslexia. For individuals with dyslexia it is especially important to know to what extent oral and silent reading are the same. One reason is that the diagnosis of dyslexia is most often based on poor oral reading fluency outcomes, whereas accommodations for the disorder usually focus on supporting silent reading. A second reason is that oral and silent reading fluency outcomes might diverge, which could mean that both reading modes should be included in diagnosis and intervention for dyslexia to be able to offer each individual the most appropriate care.

Children with dyslexia were found to read more slowly than their peers with average to good reading skills. This difference was found for both oral and silent reading. Unexpectedly, children with and without dyslexia were both found to read equally fast during oral and silent reading. In addition, oral and silent reading were found to correlate strongly with each other, as well as with a standardized measure of oral word reading. Together, these findings indicate that for children oral and silent reading fluency do not differ much, and children with dyslexia tend to be equally impaired in oral and silent reading fluency. Differences between oral and silent reading have previously been reported for an unselected sample of children of a similar age by van den Boer et al. (2014). In that study, differences between oral and silent reading were small but significant for sentence and passage reading fluency, and more pronounced for word reading, a finding running counter to the one obtained in the present study. The different findings could be due to the much larger sample size in the study by van den Boer et al. In the present study, typical readers read about 4% faster during silent than oral text reading. In the study by van den Boer et al., silent reading was about 6% faster than oral sentence reading and about 9% for passage reading. The differences between oral and silent reading thus seem of a similar magnitude, but due to a much larger sample size, the differences were significant in the study by van den Boer et al., but not in the current study. In both studies, however, the differences between oral and silent reading fluency were small.

The most likely explanation for similar reading fluency during oral and silent reading is that children in Grades 3 and 4 approach an oral and silent reading task in a similar way. This interpretation relates to the study by Prior and Welling (2001). They examined differences in comprehension after oral and silent reading for children of the same age as in the current study. Prior and Welling argue that reading is initially mainly a social experience, and only later on becomes a more private activity. When children learn to read they initially mainly read aloud to allow others to monitor and support their reading. In addition, adults read aloud to them. Only later in development, when children start to read to learn, do they start to read silently to themselves. The children in Grades 3 and 4 might read aloud either orally or in their head, resulting in no difference between reading fluency in the two reading modes.

Similar to the pattern found for the children, adolescents with dyslexia also read more slowly than their peers without reading difficulties in both oral and silent reading. However, in contrast to the pattern of the children, adolescents read faster silently than orally. This difference between oral and silent reading was somewhat smaller for adolescents with dyslexia than for typical readers. Although the correlation between oral and silent text reading was moderate, it was lower than the one found for the children. For the typical readers, the correlation of silent text reading with a standardized test of word reading fluency was low. These findings are in line with the overall finding that oral reading is slower than silent reading (e.g., Ciuffo et al., 2017; Krieber et al., 2017; McCallum et al., 2004). Due to the absence of theoretical or empirical evidence about the reasons behind differences between oral and silent reading, we can only speculate about the interpretation of this finding. Similar to previous studies, we expect that when silent reading has become the primary reading mode, readers can bypass phonological processing and access words directly through the orthographic lexicon during silent reading (e.g., Krieber et al., 2017; McCallum et al., 2004).

We also found the smaller difference between oral and silent reading for individuals with dyslexia reported by Gagliano et al. (2015), although the difference between individuals with dyslexia and typical readers was smaller in the current study. Combining the findings of Gagliano et al. (2015) with ours yields an interesting trend: With increasing reading experience, oral and silent reading seem to diverge more. For individuals with dyslexia, who lag behind in reading experience, this difference appears smaller than for their peers with average to good reading abilities. We know that fast readers on average do more leisure reading (Torppa et al., 2019) and that there is a developmental relationship between reading fluency and print exposure (van Bergen et al., 2020). These findings would agree with the interpretation that students with dyslexia read less, which might impact on silent reading fluency, via impairments in their orthographic lexicon. The orthographic lexicon is built up through decoding skills, as successful decoding of newly encountered words offers the opportunity to store these new orthographic word forms in the lexicon (e.g., Share, 1995). Compared to skilled readers, individuals with dyslexia make more errors during decoding, and read fewer words, both resulting in fewer opportunities to store orthographic representations in their lexicon. Therefore, their orthographic lexicon tends to be less extensive compared to typically developing readers (e.g. McArthur et al., 2013), thus hampering reading directly through the orthographic lexicon during silent reading and resulting in an increasing need for phonological processing, similar to oral reading. Future studies are needed to test these interpretations directly, for example by establishing direct links of phonological and orthographic processing skills with oral and silent reading.

In conclusion, the difference between oral and silent reading seems to be the same or smaller for individuals with dyslexia as compared to average to good readers. In other words, individuals with dyslexia show an impairment in silent reading that is as large as or larger than their impairment in oral reading. Furthermore, the current findings indicate that for primary school children in general and those with dyslexia in particular, oral and silent reading fluency are still the same. Therefore oral reading fluency can be an indicator of silent reading fluency, or vice versa. From adolescence onwards, however, oral and silent reading start to differ, with higher silent reading fluency as compared to oral reading fluency. Generalizations of performance or findings from oral to silent reading or vice versa are no longer justified.

It should be examined whether there are large individual differences in the difference between oral and silent reading fluency. Differences between oral and silent reading, especially if these differences vary across individuals, encourage developing valid measures of silent reading fluency for education, as well as diagnostic examinations for dyslexia. It seems important to include silent reading in diagnosing reading disabilities and dyslexia especially for adolescents. Based on an individual’s ability in both oral and silent reading it can be determined what intervention or accommodations would be most beneficial. A similar or larger deficit in silent as compared to oral reading in individuals with dyslexia justifies accommodations for the disorder that focus on silent reading, such as additional time to finish exams. Alternatively, text-to-speech software can be used to support independent silent reading when processing large amounts of text (Buzick & Stone, 2014; Wood et al, 2018). In terms of intervention, specific treatment of silent reading, in addition to oral reading, should be considered. Recent studies show promising approaches to silent reading intervention at different ages (e.g., Korinth & Fiebach, 2018; Reutzel & Juth, 2014). Interventions for silent reading seem even more important for ages outside the scope of the current study, as Ciuffo et al. (2017) found that oral reading speed remained unchanged from high school onward, but that silent reading fluency continued to increase at least up to the fifth year of university, resulting in increasing differences between oral and silent reading.

There are some limitations to the current study. Although we used an ecologically valid task of silent reading fluency, following Gagliano et al. (2015), this task did differ from our oral reading task. Whereas only reading speed was measured for oral reading, our measurement of reading fluency in silent reading included performance of some simple tasks. As a result, silent reading ability, and thereby the difference with oral reading, might have been underestimated. Including the simple tasks was done to ensure careful reading during silent reading, and to discourage skipping or inaccurate reading. However, for future studies it would be better not to include the time needed to perform these tasks in the score for silent reading. The approach of Ciuffo et al. (2017) seems more suitable. They presented their participants with a similar text including instructions, but calculated silent reading fluency based on reading time for the first part of the text, up to the first task. Unfortunately, we were not able to use the same approach in the current study as in our text only a single sentence preceded the first task. Fortunately, however, the findings of Ciuffo et al. were very similar to those of Gagliano et al., indicating that including the execution of the tasks in the score for silent reading does not seem to greatly affect the results. Alternatively, future studies could ensure equal measurement of reading ability across reading modes by selecting an appropriate measure for silent reading and administering the same task for oral reading as well (see for example van den Boer et al., 2014).

In addition, we used two age-appropriate texts to measure oral and silent reading fluency, but we did not carefully match the texts for level of difficulty, nor do we have estimates of the reliability and validity of these tasks. We tried to at least control for text difficulty by considering the number of syllables read per second as our outcome measure, rather than number of words for example. In addition, the strong correlations we found between the reading tasks, and of each reading tasks with a standardized measure of reading fluency, provide some indication that the tasks were comparable and indeed measured reading fluency. However, there is a need for replicating the current findings with adequately matched texts, as differences in difficulty level might have affected the results. The difference between oral and silent reading might have been under- or overestimated, depending on which text might be most difficult.

In sum, three main conclusions follow from this study. First, oral and silent reading fluency are comparable and even seem interchangeable for beginning readers, both for average to good readers and children with dyslexia. Second, from at least adolescence onwards oral and silent reading start to differ, indicating that both oral and silent reading should be considered in research and education, as well as the need for validated measures and developmental models of silent reading. And third, for children and adolescents with dyslexia their deficit in silent reading was as large as in oral reading or larger, justifying accommodations that focus on silent reading and encouraging studies on specific treatment of silent reading fluency. In addition, as the difference between oral and silent reading increases with age and reading experience (Ciuffo et al., 2017; Gagliano et al., 2015), continued monitoring of reading abilities in both oral and silent reading for individuals with dyslexia seems needed.
